# Misperceived pre-pregnancy body weight status predicts excessive gestational weight gain: findings from a US cohort study

**DOI:** 10.1186/1471-2393-8-54

**Published:** 2008-12-22

**Authors:** Sharon J Herring, Emily Oken, Jess Haines, Janet W Rich-Edwards, Sheryl L Rifas-Shiman, Ken P Kleinman ScD, Matthew W Gillman

**Affiliations:** 1 Obesity Prevention Program, Department of Ambulatory Care and Prevention, Harvard Medical School and Harvard Pilgrim Health Care, Boston, MA, USA; 2 Center for Obesity Research and Education, Temple University School of Medicine, Philadelphia, PA, USA; 3 Connors Center for Women's Health and Gender Biology, Brigham and Women's Hospital, Boston, MA, USA; 4 Department of Nutrition, Harvard School of Public Health, Boston, MA, USA

## Abstract

**Background:**

Excessive gestational weight gain promotes poor maternal and child health outcomes. Weight misperception is associated with weight gain in non-pregnant women, but no data exist during pregnancy. The purpose of this study was to examine the association of misperceived pre-pregnancy body weight status with excessive gestational weight gain.

**Methods:**

At study enrollment, participants in Project Viva reported weight, height, and perceived body weight status by questionnaire. Our study sample comprised 1537 women who had either normal or overweight/obese pre-pregnancy BMI. We created 2 categories of pre-pregnancy body weight status misperception: normal weight women who identified themselves as overweight ('overassessors') and overweight/obese women who identified themselves as average or underweight ('underassessors'). Women who correctly perceived their body weight status were classified as either normal weight or overweight/obese accurate assessors. We performed multivariable logistic regression to determine the odds of excessive gestational weight gain according to 1990 Institute of Medicine guidelines.

**Results:**

Of the 1029 women with normal pre-pregnancy BMI, 898 (87%) accurately perceived and 131 (13%) overassessed their weight status. 508 women were overweight/obese, of whom 438 (86%) accurately perceived and 70 (14%) underassessed their pre-pregnancy weight status. By the end of pregnancy, 823 women (54%) gained excessively. Compared with normal weight accurate assessors, the adjusted odds of excessive gestational weight gain was 2.0 (95% confidence interval [CI]: 1.3, 3.0) in normal weight overassessors, 2.9 (95% CI: 2.2, 3.9) in overweight/obese accurate assessors, and 7.6 (95% CI: 3.4, 17.0) in overweight/obese underassessors.

**Conclusion:**

Misperceived pre-pregnancy body weight status was associated with excessive gestational weight gain among both normal weight and overweight/obese women, with the greatest likelihood of excessive gain among overweight/obese underassessors. Future interventions should test the potential benefits of correcting misperception to reduce the likelihood of excessive gestational weight gain.

## Background

Over the past several decades, both actual and recommended gestational weight gains have increased in the US [1,2]. Although higher maternal weight gain was initially thought to improve infant health and reduce perinatal mortality [1,3], more recent data have focused on the negative effects of gaining too much during pregnancy [4], including undesirable birth outcomes [5-8], childhood overweight [9], and higher postpartum weight retention, which predisposes to later risk of obesity in the mother [1,3,10]. Given that approximately 40–50% of pregnant women gain more than recommended by current Institute of Medicine (IOM) guidelines [9,11,12], there is a renewed interest in better understanding the determinants of excessive gain.

Identified risk factors for excessive gestational weight gain include higher pre-pregnancy weight [13-15], primiparity [14,16], lower income [17], insufficient knowledge of weight gain recommendations (and their importance) [13], recurrent pre-pregnancy dieting [18,19], lack of health provider advice and negative attitudes about weight gain [13,20,21], along with pregnancy-related behaviors such as lower levels of physical activity and increased food consumption [17,22]. However, the relationship of a woman's perception of her weight status with weight gain in pregnancy has not been elucidated.

Among non-pregnant adolescents and young adults, evidence suggests that body weight perception is an important correlate of nutritional habits and weight gain [23-27]. Adolescents who are underweight or normal weight but perceive themselves as overweight are at a two-fold increased risk of disordered eating behaviors, including binge eating disorder or bulimia [24], which by themselves have been shown to predict the development of inappropriate weight gain and obesity [28-30]. Young adults who are overweight or obese, but do not perceive themselves as such, are less likely to engage in physical activity and weight control behaviors [25]. Without recognition of their overweight status, these individuals have been posited to ignore messages related to healthy diet and lifestyle change [26,27].

To determine whether similar relationships exist in pregnancy, we used data from a longitudinal birth cohort study to examine the association of misperceived pre-pregnancy body weight status with excessive gestational weight gain. We hypothesized that women who underassessed or overassessed their pre-pregnancy weight status would be at increased risk of excessive gestational weight gain, compared to women who accurately assessed their pre-pregnancy weight status. In addition, we explored potential mechanisms by which misperceived body weight status may promote weight gain in pregnancy.

## Methods

### Study population and design

Study subjects were recruited into Project Viva at their first prenatal visit from one of eight urban and suburban obstetric offices associated with a multispecialty group practice in eastern Massachusetts from1999 to 2002 [31]. Eligibility criteria included fluency in English, gestational age <22 weeks, and a singleton pregnancy. All mothers provided written informed consent, and all procedures were in accordance with ethical standards for human experimentation. Institutional review boards of participating sites approved the study.

Of the 2128 women who delivered a live singleton infant, we restricted our analysis to include the 1835 women who were normal weight, overweight, or obese body mass index (BMI). We did not include underweight women as they were few in number and are at low risk of excessive weight gain during pregnancy [21]. We then excluded women with missing information on perceived body weight (n = 284) or who had no measurement of gestational weight gain recorded within the 4 weeks preceding delivery (n = 14), leaving 1537 women for this analysis. Compared with the 298 women who were not included, the 1537 women in this analysis were somewhat older (mean of 32.3 versus 30.0 years), more likely to be white (72% versus 36%), college educated (68% versus 41%), and normal weight (67% versus 52%), but had only slight differences in rates of average (62% versus 64%) and overweight (37% versus 36%) pre-pregnancy weight status perception.

### Main exposure – misperceived body weight

Mothers reported pre-pregnancy weight and height by questionnaire at study enrollment. We calculated BMI (kg/m^2^) from these self-reported measures and classified women as normal weight (BMI 19.8–26.0 kg/m^2^) or overweight/obese (BMI > 26.0 kg/m^2^) according to Institute of Medicine criteria [11]. Validation of self-reported weights with clinically measured weights among a sample of 170 women in this cohort revealed a tight correlation (r = 0.99), with a mean systematic underreport of 1 kg that did not vary by maternal race/ethnicity, gestational age at enrollment into the study, or weight itself [9]. Moreover, the very high correlation indicates that ranking of individuals is well preserved.

We assessed perceived body weight status via early pregnancy questionnaire (approximately 10 weeks gestation) by asking participants, "How would you classify your weight just prior to this pregnancy?" Responses ranged from (1) markedly overweight to (5) markedly underweight. A similarly structured perceived weight status question is asked of non-pregnant women and men in the National Health and Nutrition Examination Survey [32], and has been used as a measure of weight misperception in several published reports [26,27]. Because few women in our sample had extreme weight perceptions or perceived themselves as underweight, we collapsed response categories into two groups: overweight and average/underweight.

Using BMI and perceived body weight status, we created two body weight misperception categories: normal weight women who identified themselves as overweight ('overassessors'), and overweight/obese women who identified themselves as average or underweight ('underassessors'). Women who correctly perceived their body weight status were classified as either normal weight or overweight/obese accurate assessors, and were also included in our analysis.

### Outcome – gestational weight gain

We used prenatal medical records to obtain pregnancy weights, and calculated total gestational weight gain as the difference between the last clinically recorded weight before delivery and self-reported pre-pregnancy weight. Based on current guidelines by the Institute of Medicine published in 1990 [11], we classified gestational weight gain as inadequate, adequate, or excessive. These guidelines recommend that women with a normal pre-pregnancy BMI (19.8–26.0 kg/m^2^) should gain 11.5–16 kg, that overweight women (BMI of 26.1–29.0 kg/m^2^) should gain 7.0–11.5 kg, and that obese women (BMI greater than 29.0 kg/m^2^) should gain at least 7.0 kg. As we combined overweight and obese women in our analysis, we also set an upper limit of 11.5 kg for these heaviest women [9,14]. Weight gain above recommended ranges is considered excessive. In this paper, we defined our main outcome as excessive gestational weight gain (vs. adequate or inadequate), as women who over-gain are at greatest risk for obstetric complications and postpartum obesity [1,3-10].

### Covariates

We collected information on maternal race/ethnicity, age, education, parity, employment, household income, and smoking habits, all reported by the women at their first study visit. In mid-pregnancy (approximately 26–28 weeks gestation), participants reported information about their leisure-time physical activity and diet. We were interested in evaluating mid-pregnancy behaviors as potential intermediates in the pathway between body weight misperception and excessive gain in pregnancy. Vigorous activity was of primary interest as a measure of physical activity because in this cohort we observed an association between less time spent doing vigorous activity and excessive gestational weight gain [22]. We asked each participant, "In the past 3 months, on average, how many hours per week have you spent engaged in vigorous recreational activities or sports such as jogging, swimming, cycling, aerobic dance, skiing, or other similar activities?" Our choice of examples of activities was influenced by the Physical Activity Scale for the Elderly [33], the Paffenbarger physical activity questionnaire [34], and knowledge of activities common to women in the northeastern US. Dietary intake was assessed via the semi-quantitative food frequency questionnaire (FFQ), slightly modified for use in pregnancy from the extensively validated FFQ used in the Nurses' Health Study [35,36]. We queried participants about their dietary intakes during the preceding 3 months. *A priori*, we felt it reasonable to focus on intake of fried foods away from home, fruit and vegetable consumption, and intake of a vegetarian diet [22]. We also collected data about a history of depressive symptoms prior to pregnancy with the question, "Before this pregnancy, was there ever a period of time when you were feeling depressed or down or when you lost interest in pleasurable activities most of the day, nearly every day, for at least 2 weeks," along with an indication that a professional had previously diagnosed or treated the participant for depression. We calculated gestation length from the last menstrual period, or from the second trimester ultrasound if the two estimates differed by > 10 days.

### Data analysis

We used multivariable logistic regression to examine associations between misperceived body weight and excessive gestational weight gain, using normal weight accurate assessors as the reference group. We included only those covariates that were of *a priori *interest, were independent predictors of the outcome, or confounded associations of weight misperception with excessive gestational weight gain. Our final model comprised our exposure and outcome variables, along with maternal sociodemographic factors (namely age, education, marital status, income, employment, and race/ethnicity), pre-pregnancy BMI, parity, smoking habits and gestation length. We did not find depression history or time between the last pregnancy weight and delivery to result in material changes in the magnitudes of the observed associations between misperception and excessive gestational weight gain, and therefore did not include them in the final models. Effect modification by parity (0, > = 1), race/ethnicity (white, nonwhite), and income (> or < = $40,000/year) were investigated using stratification and interaction terms (with statistical significance defined as a p < 0.05). We also explored the potential mechanisms by which misperceived body weight may promote excessive gestational weight gain through the an additional model that added mid-pregnancy behaviors and looked for attenuation of effect estimates.

Because some clinicians may use BMI categories based on WHO guidelines in practice [37,38], we performed a secondary analysis using current WHO recommendations for normal weight (BMI 18.5–24.9 kg/m^2^) and overweight/obese (BMI > = 25 kg/m^2^) to classify both misperception of body weight and categories of gestational weight gain. The results were essentially unchanged from the primary analysis (data not shown).

Given the high prevalence of excessive gestational weight gain, odds ratios (OR) are poor estimators of relative risks. For ease of interpretability, in addition to ORs and 95% confidence intervals (CI), we present risks calculated from multivariable-adjusted predicted prevalences for representative subgroups. We did this calculation by choosing covariate values corresponding to groups of interest and inverting the logit, thus back-transforming the individual predicted logits to obtain predicted probabilities [39,40]. We used SAS version 9.1 (SAS Institute, Cary, NC) to carry out all analyses.

## Results

One thousand twenty-nine (67%) of the 1537 participants reported that they were normal weight just prior to pregnancy, of whom 898 (87%) accurately perceived their weight status and 131 (13%) overassessed their weight status. Of the remaining 508 (33%) women who were overweight/obese, 438 (86%) accurately perceived and 70 (14%) underassessed their pre-pregnancy weight status. Just under one-third of participants were non-white; a similar proportion had not graduated from college. Mean age was 32.3 years (standard deviation [SD] 4.9). Compared with normal weight women who accurately perceived their pre-pregnancy weight status, overweight/obese underassessors were younger, more likely to be non-white, of lower income, less educated, and single (Table 1). During pregnancy, overweight/obese underassessors consumed fewer fruits and vegetables, but did not differ from normal weight accurate assessors in amount of vigorous activity and fried food intake. Normal weight overassessors, on the other hand, were relatively similar in all characteristics to their accurate assessor counterparts (Table 1).

**Table 1:** Distribution of characteristics by pre-pregnancy weight status perception among 1537 participants* in Project Viva

	Entire sample	Normal weight women		Overweight/obese women	
**Characteristics of participants**	n = 1537 (100%)	**Accurate assessors **n = 898 (58%)	**Overassessors **n = 131 (9%)	**Accurate assessors **n = 438 (28%)	**Underassessors **n = 70 (5%)
	*Mean (SD) or n (percent)*				
**Pre-pregnancy**					
BMI (kg/m^2^)	25.5 (5.1)	22.4 (1.6)	24.6 (1.2)	31.7 (4.9)	28.2 (2.6)
Sociodemographics					
Age (years)	32.3 (4.9)	32.3 (4.8)	33.3 (4.8)	32.5 (4.9)	30.2 (5.2)
White	1106 (72%)	691 (77%)	102 (78%)	277 (63%)	36 (51%)
College graduate	1046 (68%)	670 (75%)	97 (75%)	246 (56%)	33 (47%)
Household income ≤ $40,000	176 (12%)	81 (10%)	11 (9%)	68 (16%)	16 (28%)
Married or cohabitating	1424 (93%)	834 (93%)	125 (96%)	407 (93%)	58 (83%)
Employed	1298 (86%)	764 (86%)	103 (79%)	370 (86%)	61 (88%)
Parous	775 (50%)	403 (45%)	74 (56%)	260 (59%)	38 (54%)
History of depression	155 (12%)	85 (11%)	15 (13%)	51 (14%)	4 (7%)
Current smoker	166 (11%)	77 (9%)	15 (12%)	66 (16%)	8 (12%)

**Pregnancy**					
Behaviors					
Vegetarian diet	93 (6%)	57 (6%)	10 (8%)	21 (5%)	5 (7%)
Fried food intake away from home (servings/day)	0.1 (0.1)	0.1 (0.1)	0.1 (0.1)	0.2 (0.1)	0.1 (0.1)
Fruit and vegetable intake (servings/day)	5.9 (2.8)	6.0 (2.7)	6.0 (2.7)	5.6 (3.0)	5.3 (3.6)
Vigorous activity (hours/day)	0.1 (0.3)	0.1 (0.3)	0.1 (0.2)	0.1 (0.3)	0.1 (0.3)
Gestation length (weeks)	39.5 (1.9)	39.6 (1.9)	39.4 (1.6)	39.3 (2.0)	39.4 (2.0)
Gestational weight gain (IOM categories)					
Excessive	823 (54%)	420 (47%)	75 (57%)	271 (62%)	57 (82%)
Adequate	508 (33%)	363 (40%)	39 (30%)	98 (22%)	8 (11%)
Inadequate	206 (13%)	115 (13%)	17 (13%)	69 (16%)	5 (7%)

* We had missing data for race (<1%), marital status (<1%), education (<1%), employment (1%), vegetarian diet (2%), smoking (2%), income (7%), fried food intake (13%), fruit and vegetable intake (13%), depression history (13%), and vigorous activity (14%).

**Figure 1:**
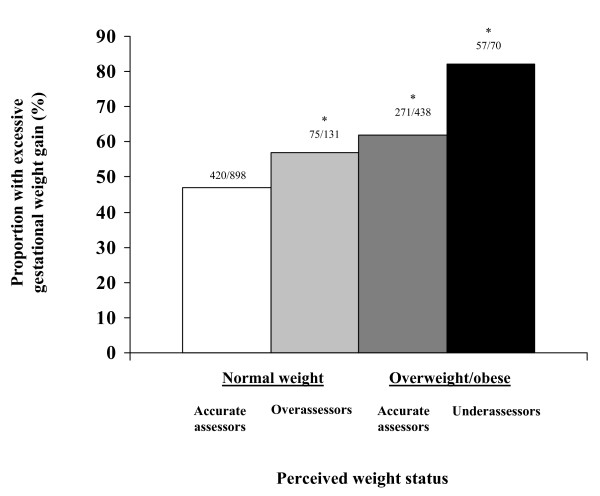
**Proportion of women with excessive gestational weight gain according to pre-pregnancy perceived weight status**. *P < 0.05 for comparisons to normal weight accurate assessors, via chi square test.

Participants gained a mean of 15.6 kg (range -7.3 to 40.9) in pregnancy (mean gestation length 39.5 weeks, SD 1.9), and 823 (54%) gained excessively according to Institute of Medicine criteria [4]. Rates of excessive weight gain varied across exposure categories: 57% of normal weight overassessors, 62% of overweight/obese accurate assessors, and 81% of overweight/obese underassessors had excessive gestational weight gain, while only 47% of normal weight accurate assessors gained excessively (p < 0.05 for all comparisons to normal weight accurate assessors, Figure 1).

On multivariable analyses, after adjustment for maternal sociodemographics, pre-pregnancy BMI, gestation length, parity and smoking habits, misperception of pre-pregnancy body weight status was associated with an increased odds of excessive gestational weight gain (normal weight overassessors, OR: 2.0, 95% CI: 1.3, 3.0; overweight/obese underassessors, OR: 7.6, 95% CI: 3.4, 17.0; Table 2). In addition, overweight/obese women who accurately perceived their body weight status had an almost 3-fold increased odds of excessive gestational weight gain compared to their normal weight counterparts. When we restricted our sample to include only the 1427 women who delivered at term (> = 37 weeks gestation), we did not observe a marked difference in the magnitude of our results (normal weight overassessors, OR: 2.1, 95% CI: 1.4, 3.1; overweight/obese accurate assessors, OR: 2.9, 95% CI: 2.2, 4.0; overweight/obese underassessors, OR: 7.8, 95% CI: 3.4, 18.0). There was no modification of the effect of weight misperception by race/ethnicity, parity, or income (all p values for interaction terms > 0.20).

**Table 2:** Odds ratios and predicted prevalences (in selected subgroups) of excessive gestational weight gain calculated from multivariable logistic regression model*

	**Pre-pregnancy weight status perception**			
	*Normal weight women*		*Overweight/obese women*	
	**Accurate assessors **n = 898	**Overassessors **n = 131	**Accurate assessors **n = 438	**Underassessors **n = 70
**Multivariable odds ratios (95% CI)**	1.0 (Referent)	2.0 (1.3, 3.0)	2.9 (2.2, 3.9)	7.6 (3.4, 17.0)

**Participant characteristics modeled†**	**Predicted prevalence of excessive gestational weight gain (prevalence ratio) **‡			
Aged 23 y, non-white, parous, lower income, less education§	37%	54% (1.5)	64% (1.7)	82% (2.2)
Aged 23 y, white, parous, lower income, less education§	45%	62% (1.4)	70% (1.6)	86% (1.9)
Aged 32 y, white, nulliparous, higher income, well-educated ||	49%	66% (1.3)	74% (1.5)	88% (1.8)

* Data from 1537 mothers participating in Project Viva.

† All groups were married, employed, non-smokers, and had mean pre-pregnancy BMI and gestation length.

‡ Prevalence ratios compare predicted prevalence in each category with normal weight accurate assessors who had the same participant characteristics.

§Household income less than or equal to $40,000 per year, some college or less.

|| Household income more than $40,000 per year, college degree.

We next studied maternal behaviors that might serve as intermediates in the pathway between weight misperception and excessive gestational weight gain. Inclusion into the multivariable model of the behaviors (vigorous activity, consumption of fried food away from home, fruit and vegetable intake, and consumption of a vegetarian diet) did not substantially alter observed associations of weight misperception with excessive gestational weight gain (OR adjusted for all factors listed previously: normal weight overassessors, OR: 2.1, 95% CI: 1.3, 3.2; overweight/obese underassessors, OR: 8.0, 95% CI: 3.2, 20.0). Therefore, we did not have evidence that these factors mediated the relationship between misperceived pre-pregnancy body weight status and excessive gestational weight gain.

Since ORs are poor estimates of relative risks when the outcome is highly prevalent, as is the case for excessive gestational weight gain, we also calculated the predicted prevalence of excessive gestational weight gain by exposure categories, using parameters in our final multivariable model. We present examples of these results in Table 2. Rates of excessive gestational weight gain exceeded 50% in all but normal weight accurate assessors, over a range of maternal sociodemographic characteristics. Comparing overweight/obese underassessors with normal weight accurate assessors, the predicted probability of excessive gestational weight gain ranged from 1.8 to 2.2. Thus, the OR of 7.6, while accurate and unbiased, overestimates the risk ratio, which is about 2.

## Discussion

In this prospective study, we found that misperceived pre-pregnancy body weight status was directly associated with excessive gestational weight gain in both normal weight and overweight/obese women. Compared with normal weight women who accurately assessed their pre-pregnancy weight status, the odds of gaining excessively during pregnancy were increased seven-fold among overweight/obese women who underassessed their pre-pregnancy body weight status. Normal weight women who overassessed their pre-pregnancy weight status had twice the odds of excessive gestational weight gain.

Our findings parallel studies in non-pregnant women that have shown associations of misperception of weight status with nutritional habits or weight gain [23-27]. To our knowledge, however, our paper is the first to report associations between weight status misperception and weight gain in pregnancy. Given both the harmful consequences and increasing prevalence of excessive gestational weight gain [1,3-10,12], identifying potentially modifiable predictors is critical to the design of interventions to reduce weight gain and improve maternal and child health.

The weight misperception variable we used could represent body dissatisfaction, often defined as the discrepancy between current and ideal body size, which may affect persons of either normal or overweight/obese weight status. Normal weight women who overassess their weight status are at increased risk of developing eating disorders, such as anorexia, bulimia and binge eating disorder [24,41]; these same disordered eating behaviors are directly related to body dissatisfaction [28,42]. Dissatisfaction with body size may also contribute to misperception among overweight/obese women who attempt to attain the media's thin ideal [28,43], promoting recurrent dieting, loss of restraint, binging, and weight gain [44]. Data linking body dissatisfaction with weight gain in pregnancy, however, are limited and inconsistent. DiPietro et al [20] reported a strong correlation between poor pregnancy body image and over-gain at 36 weeks' gestation, but more recent work has revealed an inverse relationship between body size dissatisfaction and gestational weight gain [45]. In the postpartum period, Harris and colleagues [46] found that mothers who felt more dissatisfied with their bodies immediately after pregnancy had significantly greater long term (> 2 year) weight gains than women who had no increase in dissatisfaction. More research is needed to clarify the relationships among weight status misperception, body dissatisfaction, and peripartum weight gain, given the potential for behavioral modification.

Alternatively, misperception of body weight status may signify a lack of awareness about the clinical thresholds of normal and overweight/obese. Among overweight and obese non-pregnant women, some investigators have speculated that a lack of awareness about overweight may be responsible for misperception, influenced in part by the high prevalence of the condition in the US [26]. Given that over two-thirds of Americans are overweight or obese, social comparison among overweight women might affect their judgment about their respective weight status, particularly among Black and Hispanic women for whom a heavier body image is often most accepted [25,26,47]. By failing to recognize their overweight/obese status, these women may be less likely to stay within the IOM guidelines for weight gain in pregnancy.

We speculate that a combination of biologic and behavioral mechanisms may explain the relationship between misperceived body weight and excessive gain. Recent work has implicated the prefrontal cortex, especially in the right hemisphere, as a critical area involved in the cognitive control of food intake and body size perception [46]. Whether alterations in brain function exist in women who misperceive their weight status just prior to pregnancy has yet to be fully elucidated, but provides an interesting area for further investigation. Weight misperception may also influence behaviors, such as physical activity and dietary intake, that by themselves lead to weight gain [25,26]. In our analysis, however, including dietary and activity behaviors in our models did not attenuate the relationship between misperception and excessive gain. It is unclear whether this is an issue of timing or accuracy with regard to dietary and activity assessment, or whether other behaviors not measured in our cohort, such as binge eating or dietary restraint, are the true behavioral mediators.

Also worth mentioning is our finding that overweight/obese women who accurately assess their weight status are at a 3-fold increased odds of gaining excessively during pregnancy. Correction of misperception among overweight/obese women at the start of pregnancy may therefore reduce, but not eliminate the potential for excessive gain. A better understanding of the reasons for excessive gain in these women is necessary (beyond body weight misperception), given the high proportion of overweight and obese women of childbearing age in the US.

Our study has a number of strengths including a relatively large sample size, prospective data collection, and inclusion of multiple confounding variables. However, several limitations to this study exist. Our cohort was highly educated, of higher income, and mostly white, which may limit generalizability of our results to more racially and economically diverse groups of women. In our sample, women had somewhat lower levels of misperception and overweight than have been reported elsewhere. Information about pre-pregnancy weight and weight perception was obtained via questionnaire at approximately 10 weeks' gestation and may be subject to recall bias. However, data published by Skouteris et al revealed that women "feel as fat" in early pregnancy as they did pre-pregnancy (the 3 month period prior to pregnancy), and thus weight perception is unlikely to differ substantially at the 2 timepoints [49]. It is also possible that women who misperceive do not accurately report pre-pregnancy weight; however, in a study among high school students, the proportion of students who misperceived their body weight was approximately the same regardless of whether the BMI category was calculated from measured or self-reported height and weight [23]. The weight perception and physical activity questions used here have not been validated in other pregnant populations. Diet and physical activity were measured in mid-pregnancy and may not reflect behavior in late pregnancy that could have a greater impact on gestational weight gain. As pre-pregnancy weight was self reported in our cohort, it is likely to be underestimated, and therefore gestational weight gain may be overestimated. However, our validation study indicated that ranking of individuals is preserved [9]. Finally, although the time between last measured pregnancy weight and delivery varied by up to 4 weeks in our sample, our results remained unchanged after adjustment for this difference.

## Conclusion

In summary, we found that misperceived pre-pregnancy body weight status was associated with excessive gestational weight gain. Once the etiology of the misperception variable is better understood, future interventions should test the potential benefits of correcting misperception in order to reduce the risk of excessive gestational weight gain. As women are particularly receptive to behavior change recommendations during pregnancy [50], interventions such as these may be effective in promoting long-term health among childbearing women and their children.

## Competing interests

The authors declare that they have no competing interests.

## Authors' contributions

SJH conceived of the study and participated in data interpretation and manuscript writing, under the supervision of MWG. EO, JH, and JWR contributed to the design of the study and manuscript preparation. SLR completed the analyses. KPK provided statistical support and help in interpreting findings. All authors read and approved the final manuscript.

## Pre-publication history

The pre-publication history for this paper can be accessed here:


